# A Hydrogel-Based Self-Sensing Underwater Actuator

**DOI:** 10.3390/mi13101779

**Published:** 2022-10-19

**Authors:** Shuyu Wang, Zhaojia Sun, Shuaiyang Duan, Yuliang Zhao, Xiaopeng Sha, Shifeng Yu, Lei Zuo

**Affiliations:** 1 Department of Control Engineering, Northeastern University, Qinhuangdao 066001, China; 2 Hebei Key Laboratory of Micro-Nano Precision Optical Sensing and Measurement Technology, Qinhuangdao 066004, China; 3 Department of Electrical Engineering, Chongqing University, Chongqing 400044, China; 4 Department of Mechanical Engineering, University of Michigan, Ann Arbor, MI 48109, USA

**Keywords:** hydrogel actuators, self-sensing, underwater actuators, soft-robot modelling

## Abstract

Soft robots made of hydrogels are suited for underwater exploration due to their biocompatibility and compliancy. Yet, reaching high dexterity and actuation force for hydrogel-based actuators is challenging. Meanwhile, real-time proprioception is critical for feedback control. Moreover, sensor integration to mimic living organisms remains problematic. To address these challenges, we introduce a hydrogel actuator driven by hydraulic force with a fast response (time constant 0.83 s). The highly stretchable and conductive hydrogel (1400% strain) is molded into the PneuNet shape, and two of them are further assembled symmetrically to actuate bi-directionally. Then, we demonstrate its bionic application for underwater swimming, showing 2 cm/s (0.19 BL/s) speed. Inspired by biological neuromuscular systems’ sensory motion, which unifies the sensing and actuation in a single unit, we explore the hydrogel actuator’s self-sensing capacity utilizing strain-induced resistance change. The results show that the soft actuator’s proprioception can monitor the undulation in real-time with a sensitivity of 0.2%/degree. Furthermore, we take a finite-element method and first-order differential equations to model the actuator’s bending in response to pressure. We show that such a model can precisely predict the robot’s bending response over a range of pressures. With the self-sensing actuator and the proposed model, we expect the new approach can lead to future soft robots for underwater exploration with feedback control, and the underlying mechanism of the undulation control might offer significant insights for biomimetic research.

## 1. Introduction

Unlike remotely operated vehicles that propel with substantial turbulence, soft underwater robots can enable close-up observation of aquatic life in a safe and silent way [1,2]. Especially, hydrogel-based actuators show advantages for this application [3,4] as they are compliant, wet, and biocompatible [5]. Besides, they are transparent with optic and sonic camouflage capabilities [6]. Prior hydrogel-based actuators were mostly driven by osmotic-pressure changes in response to external stimuli [7], such as temperature [8], pH [9,10], light [11,12,13], and electricity [14,15]. However, reaching high dexterity in osmotic hydrogel actuators is a grand challenge due to the coupling effect of the response time and the force [16]. Therefore, hydraulic and pneumatic actuations are introduced to propel the hydrogel-based actuators [17,18]. When the actuator bends bidirectionally, mimicking a fish’s caudal fin oscillation, the movement shows high energy efficiency similar to natural swimming animals [19]. Further insights into the internal dynamics of the soft structure may lead to an improved understanding of the underlying locomotion physics. 

Meanwhile, practical applications of soft actuators typically require feedback control to adjust the body’s movement [20,21,22]. In this way, the robotic fish can match the frequency of body undulation with the incident flow to swim efficiently, and therefore sensing devices to monitor the bending are necessary [23,24]. Currently, embedding soft sensors for position, pressure, and tactile sensing is an essential trend in soft robotics [25,26,27]. However, the fabrication process of soft robots may be complicated by the introduced sensors [28,29]. Additionally, due to the unavoidable differences in the physical properties of the sensor and actuator, the accuracy of the measurement may downgrade. 

A viable approach to solve the issue is by constructing intelligent soft materials that simultaneously actuate and sense the motions similar to living organisms [30,31]. Living organisms perceive and control the motion to adapt to the environment by sensing their movement to provide somatosensory feedback for the neuromuscular systems [32]. A similar self-sensing approach has been reported using the ionic polymer–metal composite [33,34], the carbonaceous polymer [24], and conductive fluid [35]. Yet some of them did not show sufficient elasticity for soft-robot applications. Additionally, hydrogel-based self-sensing remains scarcely explored.

When designing control systems for bioinspired systems, modeling is another critical issue to consider alongside sensing feedback since the soft robots are typically underactuated and show material nonlinearity. An accurate model may also benefit the computational optimization and algorithms for thrust production. Previously, constant curvature [36], the Cosserat-rod-theory-based model [37], and the machine-learning-based approaches [38] have been demonstrated under the steady-state assumption. However, these methods are computationally intensive, and thus the reduced-order dynamic model with simplification is essential for easy implementation.

Herein, we introduce an underwater hydrogel-based actuator inspired by biological neuromuscular systems. It is fabricated by polyacrylamide (PAAm) and N-Methylenebisacrylamide (MBAA) with high stretchability. We assembled the two PneuNet-shaped chambers with adhesives to enable bi-directional actuation and applied hydraulic power to achieve high dexterity and force. Then, we utilized the strain-induced resistance change for self-sensing and demonstrated that it could accurately estimate the bending of the ionic actuators in real-time. Meanwhile, we proposed a simple first-order system modeling method using the experimental data for predicting bending angle dynamically. It could predict the system’s motion in a computationally light way. With the proposed self-sensing proprioception and modeling technique, the method might pave the way for future underwater soft robots with closed-loop controls. Further insights into the underlying mechanisms in soft actuators’ fluid control may potentially offer biomimetic technology transfer.

## 2. Materials and Methods

### 2.1. Materials 

The acrylamide (AAm) was used as the monomer for the covalently cross-linked network in the tough hydrogel. In the polyacrylamide (PAAm) network, N-Methylenebisacrylamide (MBAA) was used as the crosslinker and N, N, N′, N′-Tetramethylethylenediamine was used as a catalyst for polymerization. Ammonium persulfate (AP) was used as the initiator for polymerization. Sodium chloride was added to the hydrogel to enhance the electrical conductivity. To release the hydrogel from the mold, we used a release agent (Ease Release 200). To bond hydrogel chambers, we used 1-Octadecene and instant adhesive (Loctite 406). All the chemicals were purchased off the shelf edand used directly without further purification. As hydraulic connections, various sizes of silicone tubings were used.

### 2.2. Fabrication of Hydraulic Hydrogel Actuators

**Figure 1:**
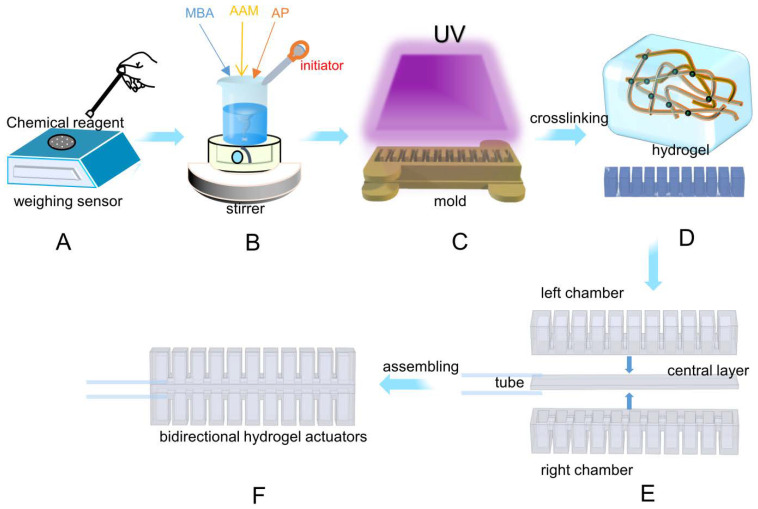
The fabrication process of the bi-directional bending actuator. (**A**,**B**) The process of preparing precursor liquid. (**C**,**D**) We pour the precursor into the prepared mold at night for cross-linking curing under UV light. (**E**,**F**) We get two symmetric chambers and an intermediate layer after mold release. We glue them with binder and assemble them into a bidirectional bending actuator.

The hydraulic hydrogel actuators’ molds were fabricated using a 3D printer and laser cutter based on computer-aided design modeling. As shown in Figure 1, we added the weighed reagents into deionized water in sequence and stirred it for one hour to form the evenly mixed pre-gel solution. Then, the pre-gel solution was poured into the mold treated with a release agent after degassing. The pre-gel was solidified by heating at 40 °C for 20 min under the 364 nm ultraviolet lamp, followed by naturally cooling down to room temperature. 

The actuator is composed of two chambers, a central layer, and elastomer tubes. They are assembled by bonding using the adhesive, composed of 1-Octadecene and Loctite 406(1:4), to form the bidirectional actuators. Before underwater experiments, a layer of silicone protective layer is applied on the surface of the hydrogel.

### 2.3. Mechanical Properties and Test Methods of Hydrogels

To perform the tensile test, the hydrogel samples were prepared in cubic shape (1.5 × 1 × 0.5 cm). To prevent dehydration during the test, the surface of the hydrogel was coated with glycerin before being mounted to the fixture. When the samples were stretched at a constant speed, the force was measured in real time by a push-pull gauge (HANDPI HP-2), and the stretched length was recorded accordingly.

### 2.4. Experimental Platform Setup

**Figure 2:**
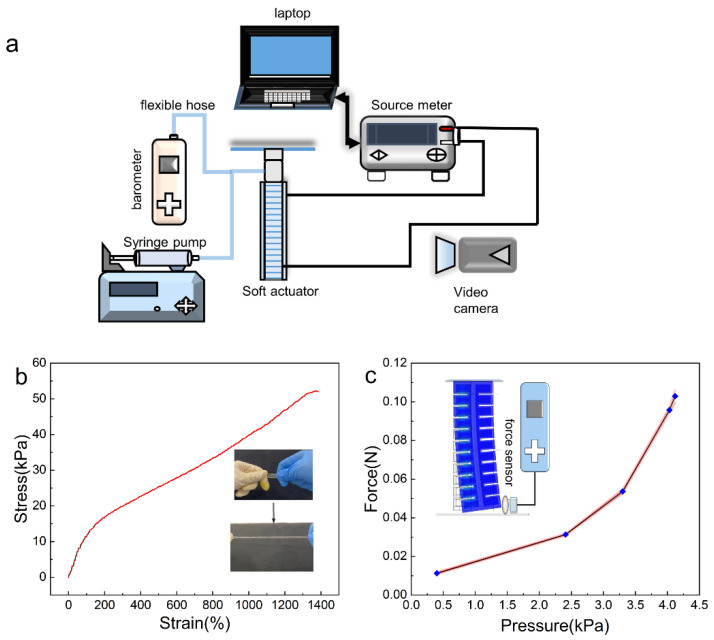
(**a**) The schematic diagram of the experimental setup. (**b**) The stress–strain curve of the hydrogel. (**c**) The generated force of the actuator at different pressures.

In Figure 2a, we show the schematic view of the experimental platform. The resistance of the hydrogels was measured by a sourcemeter (Keithley 2400), and the data were collected by the LabView program on a laptop. We electrically connect the hydrogels to the sourcemeter’s cable by conductive fabric to avoid the Schottky barrier. The hydrogel actuator itself was connected in a series as a complete resistor in the test circuit, with electrodes positioned at both ends of the actuator.

The actuator can be actuated hydraulically or pneumatically by a programmed syringe pump. The pressure in the chamber was recorded by a barometer, and the bending angle of the soft actuator was recorded by a camera. The force generated by bending of the actuator was recorded by a force gauge (HANDPI HP-2, Figure 2c).

### 2.5. Finite-Element Analysis (FEA) Simulation

To investigate the stress distribution and bending effect of the hydrogel actuator, we built a 3D finite element model using Comsol software based on PneuNet’s geometry. The two imported cavities were combined with a substrate to form the bi-directional bending actuator. Subsequently, the union was meshed into tetrahedral elements. The Ogden model is most suitable for dealing with large strain problems and has higher simulation accuracy. To capture the elastic response of the actuators, we modeled both hydraulic chambers and the stiff layer as a hyper-elastic solid using the incompressible Ogden model [4]. The strain energy potential function is as follows:

(1) $$W_{siso}=\overset{N}{\underset{i=1}{\sum }}\frac{\mu_{i}}{\alpha_{i}}\left({\bar{\lambda_{e1}}}^{\alpha_{i}}+{\bar{\lambda_{e2}}}^{\alpha_{i}}+{\bar{\lambda_{e3}}}^{\alpha_{i}}-3\right)$$

where N is the order of the model, and usually N is taken to be a number between 1 and 3. $\alpha_{i}$ and $\mu_{i}$ are the intrinsic material constants. $\lambda_{e1}$, $\lambda_{e2}$, and $\lambda_{e3}$ are the strains in the three orthogonal anisotropic directions. The material parameters are identified as μ = 20.927 kPa, α = 2.5252. The self-contact interaction characteristics are set on all outer surfaces and inner walls of the actuator to simulate the interaction conditions of the surface under pressure. The experimental boundary conditions are applied by constraining one side of the actuator, and the load is applied to the inner wall of the cavity to simulate the pressure. Iterative operations are performed by a steady-state solver with a step size set to 0.1 s. The bending of the actuator is dynamically displayed by animation. The stress–strain relationship is characterized by the color legend. 

## 3. Results

### 3.1. Characteristics of Actuation

To ensure that the hydrogel can endure the deformation during actuation, the elasticity of the hydrogel was validated experimentally by tensile test, and the stress–strain curves of the hydrogel are plotted in Figure 2b. In the tensile experiment, the strain of the hydrogel could reach up to 1400% and its stress could reach up to 50 kPa without cracks. Therefore, the selected material showed high toughness and elasticity.

We first measured the hydrogel actuator’s force perpendicular to the body. The maximum force can reach 0.1 N at a pressure of 4 kPa (Figure 2c). This enables the bi-directional hydrogel actuator to move forward swiftly when undulating periodically. We also find the actuator’s force increases faster at higher pressure.

**Figure 3:**
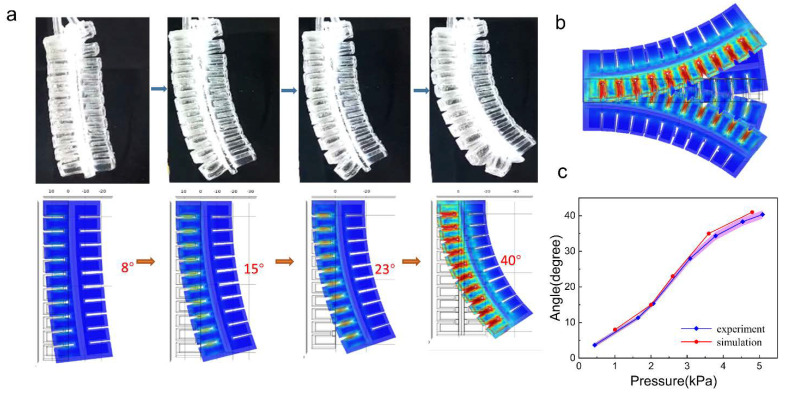
(**a**) The bending effect of the actuator at different angles in experiments and simulations. (**b**) Simulated effects of the actuator bending bidirectionally. (**c**) The bending angles of the actuator under different pressures in experiments and simulations.

Subsequently, we investigated the bending behavior of the actuator by simulation and experiment. We showed the bending behavior of the actuator in simulation and experiment at four discrete angles (Figure 3a). By summarizing the angle and the corresponding pressure, we plot the results in Figure 3c. We find the experimental results are consistent with those of the FEA simulation. Since the actuator is constructed symmetrically, the actuator can bend bidirectionally (Figure 3b). Such simulation results can be used to guide the design and optimization of geometry parameters for improved bending ability in future work. We provide them in the Supplementary Material.

**Figure 4:**
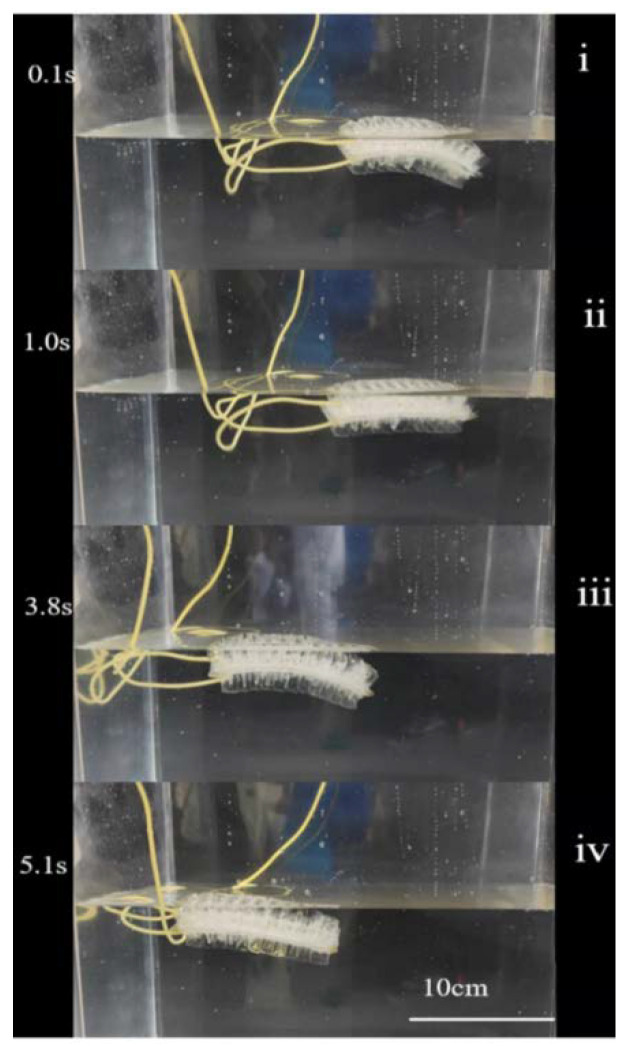
Images of the actuator moving in water in a time sequence of i–iv.

Then, we demonstrated the actuator’s application for underwater swimming. Figure 4 shows a series of pictures of the bidirectional bending soft actuator swimming in water. At a driving frequency of 1 Hz, the actuator’s swimming speed is around 0.19 BL/s. Further optimization of the geometry based on FEA and the algorithm might improve the performance. Several typical soft-robot fish are shown in Table 1, in which their materials, actuations, swimming, and advantages are compared. In the future work, we expect to equip the soft actuator with a tin filled with pressured gas and mechanical controlled valves to regulate the pressure for untethered actuation.

### 3.2. Self-Sensing Characterization

After adding conductive ions to the hydrogel, the conductivity of the hydrogel is significantly enhanced, and the actuator made of hydrogel can be a resistive sensor upon stretching. The strain-induced resistance change of the conductive hydrogel is attributed to the structural effect involving elongation along the tensile direction and Poisson contraction along the lateral direction.

**Figure 5:**
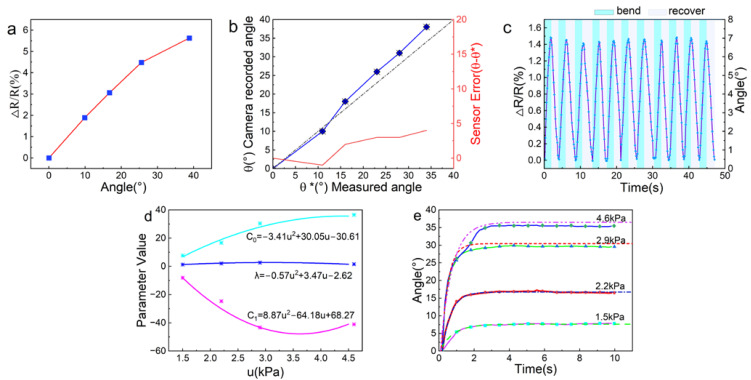
(**a**) The relationships between ΔR/R and angles. (**b**) The actuator’s self-sensing angle versus the camera’s recorded angle. The asterisks represent the actuator’s self-sensing angles and the camera’s recorded angles under different pressure. The sensor’s error is also provided by subtracting the measured difference and is indicated by the solid red line. The dotted line represents angular consistency under ideal conditions. (**c**) The curves show the change of resistance and angles of the actuator during cycles of bending and recovery. (**d**) Fitted parameters versus pressures. (**e**) Comparison of the model’s prediction and the step responses of the soft actuator subjected to different input pressures. The dotted lines represent the model’s prediction. The solid lines indicate the experimental data.

We then measure the actuator’s resistance during the bending process and correlate it to the bending angles for calibration. The relationship between the change of resistance and the bending angle of the actuator is illustrated in Figure 5a. The sensitivity is around 0.2%/degree when the bending angle is within 25°. 

Next, we compare the self-sensing angle and the actual bending angle recorded from the camera to validate the sensing accuracy. As the angle increases, the value of the sensing error increases, but the angle error is within 15% (Figure 5b). A possible reason for the error might be the material’s inherent hysteresis when actuating. Further improvement is expected with the tuned hydrogel’s composition for lower hysteresis.

Additionally, we show the resistivity and angle as a function of time as it bends and recovers (Figure 5c). We demonstrate that it can capture the actuator’s deflection in real-time. Additionally, the rate of resistance change and the bending angle of the actuator can maintain good consistency over multiple cycles. These demonstrate that the hydrogel soft actuator’s self-sensing can monitor the motion in real-time.

### 3.3. Dynamic System Modeling

To model the soft actuator’s dynamics with high accuracy is challenging due to the high dimensional deformation. Due to the high damping and low inertia properties of soft robots, we propose to model the soft actuator’s dynamics using first-order differential equations. The dynamic model of the soft actuator is modeled as follows:

(2) $$\dot{\theta }\left(t\right)+\lambda \theta \left(t\right)=C_{0}\lambda$$

where θ(t) is the bending angle of the actuator varied with time. The solution to Equation (2) is in the form of

(3) $$\theta \left(t\right)=C_{1}e^{-\lambda t}+C_{0}$$

The response of the system can be fitted by the time domain equation. The parameters of the step response curves under different pressures are different, but they are all related to the pressure. We fit the parameters by least-square fitting into a function related to pressure u. The corresponding values of the parameters $C_{1}$, $C_{0}$, and λ are determined by the specified pressure u and the fitting equations:

(4) $$\begin{array}{c} C_{0}=a_{2}u^{2}+a_{1}u+a_{0}\\ C_{1}=b_{2}u^{2}+b_{1}u+b_{0}\\ \lambda =c_{2}u^{2}+c_{1}u+c_{0} \end{array}$$

where $a_{i}$,$\;b_{i}$,$\;c_{i\;}$(i=0,1,2) are constant coefficients derived from the step response experiment, and the fitted parameters under different pressures are shown in Figure 5d.

To examine the motion prediction of proposed dynamic model, step response experiments were conducted. When applying different pressures as the input, the step responses of actuator were monitored. Then, the model predicted results are compared with the experimental data from 2.2 kPa to 4.6 kPa (Figure 5e). Here, the recorded angles are obtained from the self-sensing data. As can be seen from the curves in the figure, the model predictions are very close to the experimental data, with a maximum error of 3.67%. This demonstrates the feasibility of the first-order dynamic model for modeling our soft actuator. In addition, we can extract the time constant from the step response to be 0.83 s, indicating the system fast response for dexterous swimming.

## 4. Conclusions

In this paper, we designed, fabricated, modeled, and characterized a self-sensing bi-directional bending underwater actuator. Using the PAAm-and-MBAA-based stretchable hydrogel, a soft actuator was fabricated by assembling two PneuNets symmetrically. It was hydraulically actuated to undulate with high force (0.1 N) and a fast response time (time constant 0.83 s). The actuation performance was then demonstrated for underwater swimming with a speed of 2 cm/s (0.19 BL/s). To characterize the actuator bending behavior, we simulated the actuator under various pressures with FEA and verified it with experimental data. Subsequently, using the relationship between the change of resistance and the bending angle, we managed to couple the soft actuator with the sensor for real-time angle monitoring with high accuracy. The proprioception sensitivity reached 0.2%/degree. In addition, we also modeled the actuator dynamics with a first-order differential equation using the experimental data, which accurately predicted the actuator’s bending motion dynamically in a computationally light way. In summary, the modeling and the self-sensing technique can enable feedback control for future endeavors. Further development of this self-sensing actuator might contribute to the next generation of soft robots for underwater exploration.

## Figures and Tables

**Table 1:** Comparisons of recently shown underwater soft actuators. The speed is described using body length/second (BL/s).

Actuator Shape	Materials	Actuation Method	Speed and Frequency	Feedback Sensing	Modeling	Reference
Fish	Hydrogel	Hydraulic	0.19 BL/s (1 Hz)	No	FEA	[6]
Fish	Dielectric elastomer	Voltage induced stress	0.24 BL/s (1 Hz)	No	FEA	[4]
Jelly-fish	Hydrogel	Pneumatic	0.65 BL/s (0.67 Hz)	No	FEA and linear approximation	[39]
Fish	Hydrogel	Electroresponse	0.006 BL/s (0.3 Hz)	No	NA	[40]
Jelly-fish	Liquid metal	Electromagnetic response	0.2 BL/s (1 Hz)	Yes	NA	[41]
Leptocephali	Dielectric elastomer	Voltage induced stress	0.009 BL/s (0.33–0.5 Hz)	No	Analytical bending model	[42]
Octopus	Hydrogel	Photothermal response	34.6°/s	Yes	NA	[30]
Fish	Silicone, eGaIn	Pneumatic	0.8–1.2 Hz	Yes	data-driven lumped parameter model	[19]
Fish	Hydrogel	Hydraulic	0.18 BL/s (1 Hz)	Yes	Differential equation and FEA	This work

## Data Availability

The data that support the findings of this study are available from the corresponding author upon reasonable request.

## Supplementary Materials

The following supporting information can be downloaded at: https://www.mdpi.com/article/10.3390/mi13101779/s1, Video S1.
